# Al_x_In_1−x_N on Si (100) Solar Cells (x = 0–0.56) Deposited by RF Sputtering

**DOI:** 10.3390/ma13102336

**Published:** 2020-05-19

**Authors:** Sirona Valdueza-Felip, Rodrigo Blasco, Javier Olea, Alba Díaz-Lobo, Alejandro F. Braña, Fernando B. Naranjo

**Affiliations:** 1 Photonics Engineering Group, University of Alcalá, 28871 Alcalá de Henares, Spain; rodrigo.blasco@uah.es (R.B.); fernando.naranjo@uah.es (F.B.N.); 2 Thin Film and Microelectronics Group, University Complutense of Madrid, 28040 Madrid, Spain; oleaariza@fis.ucm.es; 3 Electronics and Semiconductors Group, Applied Physics Department, Universidad Autónoma de Madrid, 28049 Madrid, Spain; alba.diaz@uam.es (A.D.-L.); alejandro.brana@uam.es (A.F.B.)

**Keywords:** AlInN, silicon, sputtering, solar cells

## Abstract

We investigate the photovoltaic performance of solar cells based on n-Al_x_In_1−x_N (x = 0–0.56) on p-Si (100) hetero-junctions deposited by radio frequency sputtering. The Al_x_In_1−x_N layers own an optical bandgap absorption edge tuneable from 1.73 eV to 2.56 eV within the Al content range. This increase of Al content results in more resistive layers (≈10^−4^–1 Ω·cm) while the residual carrier concentration drops from ~10^21^ to ~10^19^ cm^−3^. As a result, the top n-contact resistance varies from ≈10^−1^ to 1 MΩ for InN to Al_0.56_In_0.44_N-based devices, respectively. Best results are obtained for devices with 28% Al that exhibit a broad external quantum efficiency covering the full solar spectrum with a maximum of 80% at 750 nm, an open-circuit voltage of 0.39 V, a short-circuit current density of 17.1 mA/cm^2^ and a conversion efficiency of 2.12% under air mass 1.5 global (AM1.5G) illumination (1 sun), rendering them promising for novel low-cost III-nitride on Si photovoltaic devices. For Al contents above 28%, the electrical performance of the structures lessens due to the high top-contact resistivity.

## 1. Introduction

Nowadays, the main goal of the photovoltaic industry is to develop novel technologies that manage to simultaneously improve device efficiency and reduce fabrication costs. In that sense, materials such as perovskites [1] and III-V compounds [2] have obtained great results. However, new low-cost materials are needed to complement the already established Si technology. III-nitride semiconductors and alloys are very promising for application in solar cells because of their tunable wide direct bandgap energy from the near-infrared (0.7 eV, InN) to the ultraviolet (6.2 eV, AlN) and their particular material properties, such as thermal and chemical stability, make them quite promising to be used in space applications [3].

The difficulties of growing high-quality and single-phase Al_x_In_1−x_N relies on the big differences between its binary constituents, InN and AlN (bonding energies, lattice mismatch, and growth temperature) being responsible for the immiscibility gap, and therefore for the phase separation and composition inhomogeneities commonly present in the alloy [4].

The growth of Al_x_In_1−x_N alloys has been reported by different techniques, but mainly by metal-organic chemical vapour deposition [5,6,7], molecular beam epitaxy [8,9,10], and sputtering [11,12,13]. The first two growth techniques achieve the fabrication of high crystal quality layers at high growth temperatures. However, radio frequency (RF) sputtering presents some extra advantages since this technique permits the deposition of polycrystalline single-phase Al_x_In_1−x_N on large-area substrates using a low-cost technology exportable to the industry.

Our research group already has experience concerning the growth of Indium rich Al_x_In_1−x_N (x~0–0.35) on different substrates, such as glass [14], sapphire [15], and Si (111) with [16] and without [17] an AlN buffer layer. Sputtered Al_x_In_1−x_N can be successfully deposited in a low-temperature regime, ranging from room temperature to 550 °C. We have synthesized nitride layers on sapphire with high structural quality, bandgap energy from 1.7 eV to 2.0 eV, and a photoluminescence emission at room temperature that blue shifts from 1.59 eV (InN) to 1.86 eV (Al_0.36_In_0.64_N) depending on the Al mole fraction [15].

III-nitride materials are usually grown on Si substrates with a (111) cubic crystal orientation due to the lower lattice parameter mismatch between the wurtzite nitride structure and the silicon one. However, silicon-based solar cells are based on silicon with (100) cubic crystal orientation due to their lower density of atoms at the surface and of dangling bonds that generate non-desired recombination centres [18]. So, to accommodate III-nitrides to this technology it is required the development of Al_x_In_1−x_N layers on Si (100) substrates. In that sense, InN and Al_x_In_1−x_N layers have been fabricated by molecular beam epitaxy methods on Si (100) [19,20], achieving layers oriented along the c-axis. Nevertheless, the crystalline quality of the Al_x_In_1−x_N (x = 0.20–0.43) material degrades for Al contents above x > 0.36 because of the presence of structural defects [20]. He et al. reported the properties of InN and Al_x_In_1−x_N (x < 0.30) layers deposited on Si (100) by RF magnetron sputtering, getting also layers oriented along the c-axis and without phase separation [21]. Also, Afzal et al. studied the fabrication of Al_x_In_1−x_N thin films on Si (100) through the deposition of single stack InN films by reactive RF magnetron sputtering in an Ar–N_2_ environment and then the deposition of an Al stack by direct current sputtering in Ar atmosphere at room temperature. To get wurtzite crystalline layers, annealing of the layers at 400 °C for at least 4 hours was needed [22].

On the other hand, Liu et al. reported first solar cells based on n-Al_x_In_1−x_N (x = 0.27) on p-Si (100) hetero-junctions deposited by RF sputtering, pointing to a conversion efficiency of 1.1% under AM-1.5G illumination (1 sun) for bandgap energy of 2.1 eV [23]. We have also recently reported the influence of including an AlN interlayer thickness (0–15 nm) on the photovoltaic performance of Al_x_In_1−x_N (x = 0.37) on Si (111) hetero-junctions obtaining best results—a conversion efficiency of 1.5% under 1-sun AM1.5G illumination [24]—for devices with a 4-nm-thick AlN buffer layer, thanks to the improvement of the Al_x_In_1−x_N crystalline quality [16]. For thicker buffers, the performance is reduced due to inefficient tunnel transport through the AlN buffer.

These are the first efficiency reports of Al_x_In_1−x_N-based solar cells deposited by sputtering. They are quite promising but need much improvement. For example, these devices present “S” shaped current density-voltage curves under illumination, which strongly limit their performance reducing the fill factor to 20% probably due to the presence of interfacial defects. To get rid of this double diode characteristic of the junction, we studied the effect of increasing the substrate temperature so that the mobility of the adatoms at the growing surface would be high enough to reduce the density of the non-desired recombination centres [25]. Electrical results on Al_x_In_1−x_N (x = 0.40) on Si (111) layers deposited at 550 °C point to a current density-voltage response without "S" shape, while maintaining the high Al_x_In_1−x_N crystal quality achieving record conversion efficiency values for this technology of 2.5% [26].

This is a very good step forward, but the fabrication of efficient Al_x_In_1−x_N on Si devices needs the increase of the Al mole fraction of the alloy to increase the overlapping of the device spectral response with the maximum of the solar spectrum and therefore increase their conversion efficiency. Recent theoretical simulations of the photovoltaic characteristics of this kind of hetero-junction forecast their potential as solar cells estimating a maximum conversion efficiency of 23.6% in devices with Al mole fractions ~0.50 and including an antireflection coating [27].

In this paper, we report the electrical and optical properties of n-Al_x_In_1-x_N on p-Si (100) hetero-junctions deposited at 550 °C by RF magnetron sputtering as a function of the Al mole fraction (x = 0–0.56) with the propose of developing operative low-cost solar cells. The photovoltaic performance of these junctions is also studied and deeply analysed.

## 2. Materials and Methods

### 2.1. Al_x_In_1−x_N Deposition by RF Sputtering

Al_x_In_1−x_N samples were deposited on p-doped 300-μm-thick Si (100) with a resistivity of 1–10 Ω·cm using a reactive RF magnetron sputtering system with 2 inch confocal magnetron cathodes of pure indium (4N5) and pure aluminium (5N), and pure nitrogen (6N) as the reactive gas. The substrates were chemically cleaned in organic solvents before being loaded into the chamber where they were outgassed at 600 °C for 30 min. Then, substrates were cooled down to the growth temperature. Before deposition, targets and substrate were cleaned using a plasma etching with Ar (5N) in the growth chamber. Optimized Al_x_In_1−x_N layers present in this study were deposited with a nitrogen flow of 14 sccm at a pressure of 0.47 Pa. The RF power applied to Al target, P_Al_, was varied from 0 to 225 W (samples D1 to D7 in Table 1) to modulate the Al mole fraction, while the RF power applied to In target and the temperature were fixed to 30 W and 550 °C, respectively. Under these deposition conditions the deposition rate increases linearly with P_Al_ from 0.59 nm/min for P_Al_ = 0 W to 1.66 nm/min for P_Al_ = 225 W. Taking this in mind, we have adjusted the deposition time to target a nominal thickness of the Al_x_In_1−x_N layer of 90 nm. X-ray reflection (XRR) measurements performed in these films point to an average thickness of the Al_x_In_1−x_N of 88 ± 8 nm. More details about the deposition procedure and characterization of the samples can be found in ref. [28].

### 2.2. Material Characterization

The electrical and optical properties were analysed in Al_x_In_1−x_N layers deposited on sapphire substrates at the same time and under the same growth conditions. We used room-temperature Hall-effect measurements under the conventional van der Pauw geometry. The optical properties of the layers were estimated from transmittance measurements carried out at normal incidence using an optical spectrum analyser over the 350–1700 nm wavelength range at room temperature.

### 2.3. Device Fabrication

**Figure 1:**
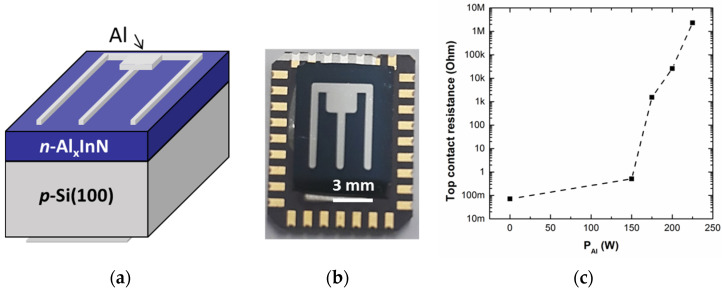
(**a**) Schematic description of an Al_x_In_1−x_N on Si hetero-junction solar cell. (**b**) Top-view image of a representative device with ~0.7 cm^2^ area. (**c**) Variation of the top n-contact resistance deposited on the Al_x_In_1−x_N layer vs the power applied to the Al target (P_Al_) used for the Al_x_In_1−x_N growth.

Al_x_In_1−x_N on Si structures were processed into devices of ~0.7 cm^2^, with both top and bottom contacts formed by 100 nm-thick Al deposited by RF sputtering. Before the deposition of the Al_x_In_1−x_N film, the bottom contact to p-Si was sputtered and then annealed at 450 °C during 3 min under nitrogen atmosphere to provide an ohmic behaviour [25]. Afterwards, Al_x_In_1−x_N films were fabricated and finally, the top n-contact was deposited. Figure 1a,b show a schematic description of the Al_x_In_1−x_N on Si solar cell structure with a top-view optical microscopy image of a representative device, respectively.

The resistivity of the top and bottom contacts was obtained from transmission line measurements on Al_x_In_1−x_N on sapphire samples and bare p-Si substrates, respectively. The ohmicity of the n-Al_x_In_1−x_N top contact was evaluated measuring the current density-voltage curve between two Al pads (2.5 × 1 mm^2^) spaced from 0.5 to 3 mm in the dark; while the ohmicity of the p-Si bottom contact was evaluated in bare p-Si wafers measuring the current density-voltage curve between two Al pads (1 × 0.5 cm^2^) spaced 1–6 mm in the dark [25]. Figure 1c presents the evolution of the n-contact resistance as a function of P_Al_ used for the Al_x_In_1−x_N growth, which increases from 0.07 Ω to 2.2 MΩ in the studied range. The bottom p-contact resistivity is ~6 Ω·cm^2^.

### 2.4. Device Characterization Techniques

Devices were characterized by current density-voltage curves performed in dark and under standard solar illumination at 25 °C. Dark measurements were carried out with a four-point probe station, whereas measurements under illumination were recorded in a solar simulator with an AM1.5G spectrum under 1 sun (P_in_ = 100 mW/cm^2^). External quantum efficiency (EQE) measurements were carried out at room temperature exciting with a 250 W halogen lamp coupled to an Oriel Cornerstone 130 1/8 m monochromator in the visible range (400–1100 nm) at zero bias. A red He-Ne laser (λ = 633 nm) with an output power of 0.6 mW was used to calibrate the generated photocurrent versus the input optical power.

## 3. Results and Discussion

The structural, morphological and optical quality and properties of the Al_x_In_1−x_N on Si (100) layers understudy was deeply analysed in reference [28]. In this report, our group demonstrated the successful deposition of high-quality and wurtzite single-phase n-type Al_x_In_1−x_N layers with an Al mole fraction over the range of x = 0 to x = 0.56 on p-Si (100) substrates by RF magnetron sputtering. High-resolution X-ray diffraction measurements revealed that the layers own wurtzite single-phase crystalline structure oriented along the c-axis perpendicular to the sample surface. 2θ/ω scans performed in these layers show the diffraction peaks associated to the Si (100) and the AlInN (0002) reflections revealing that there is no phase separation in the AlInN alloy along the studied range of Al power supply and that no other parasitic reflections related to other phases were detected. On the other hand, the full width at half maximum of the rocking curve around the Al_x_In_1−x_N (0002) diffraction peak decreased from ~9° to ~3° when incorporating Al to the Al_x_In_1−x_N alloy. The root mean square surface roughness of the samples, estimated from atomic force microscopy, evolved from 20 nm for InN to 1.5 nm for Al_0.56_In_0.44_N films. For Al mole fractions equal and below 0.35, samples showed strong low- and room-temperature photoluminescence emission, which blue-shifted from 1.59 eV for InN to 1.82 eV for Al_0.35_In_0.65_N samples at 11 K. Taking into account these results, the developed n-Al_x_In_1−x_N/p-Si junctions presented promising material properties to explore their performance operating as solar cells.

**Figure 2:**
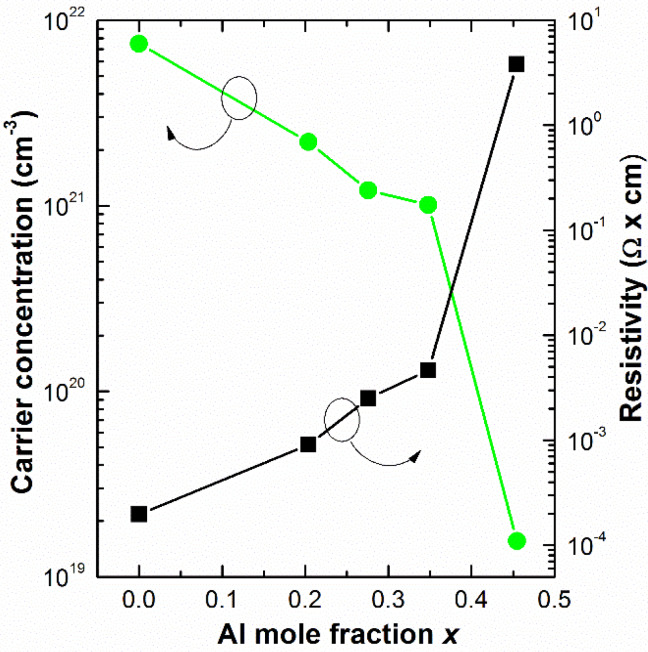
Dependence of the room temperature carrier concentration and resistivity of the Al_x_In_1−x_N on sapphire films vs the Al mole fraction x. For x > 0.45 electrical measurements were not reliable due to the high resistivity of the layers, being above the resolution of the Hall effect setup.

To develop efficient devices, it is of crucial importance to proper control the n-type doping. For this aim, Hall effect measurements were carried out in Al_x_In_1−x_N films deposited on sapphire at the same time and under the same growth conditions to explore the evolution of their electrical characteristics, such as carrier concentration, layer resistivity, and carrier mobility, depending on the alloy mole fraction. As plotted in Figure 2, the resistivity of the III-nitride layers increases four orders of magnitude from 0.2 mΩ·cm to 3.4 Ω·cm for the InN (D1) to the Al_0.45_In_0.55_N samples (D5), respectively. We would like to point out that AlN material usually owns a resistivity higher than 10^14^ Ω·cm [29]. In this sense, the residual n-type carrier concentration decreases by almost three orders of magnitude from 7.5 × 10^21^ cm^−3^ to 1.6 × 10^19^ cm^−3^, and the carrier mobility decreases from 4.2 cm^2^/V·s to 0.1 cm^2^/V·s within the same Al mole fraction range. The responsible for the high carrier concentration of these layers are the unintentional doping coming from hydrogen and/or oxygen impurities incorporated during the sputtering deposition, and point defects like nitrogen vacancies, as previously reported [30,31,32].

**Figure 3:**
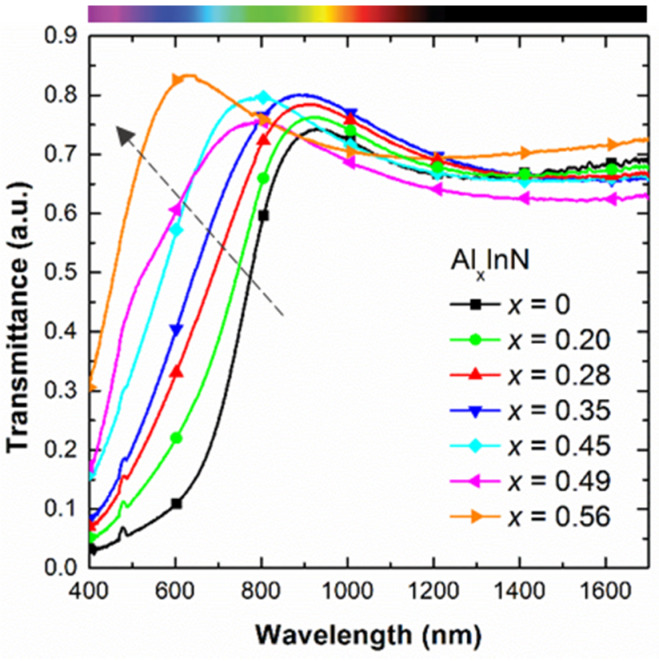
Optical transmission spectra of the Al_x_In_1−x_N on sapphire samples vs the Al mole fraction.

Figure 3 shows the optical transmittance spectra measured in Al_x_In_1−x_N samples deposited simultaneously on sapphire under the same deposition conditions vs the Al mole fraction x. The optical absorption band-edge blue shifts as expected with the Al content. However, transmittance measurements of layers with thickness below 100 nm never reach zero values at short wavelengths. This effect can lead to an overestimation of the estimated bandgap energy.

To overcome this, we estimated the bandgap energy from transmittance spectra measured in thick Al_x_In_1−x_N on sapphire samples (thicknesses above 300 nm) with an Al mole fraction similar to those of the samples of this paper grown at 300 °C by RF sputtering [25]. The method to estimate the bandgap energy from transmittance measurements is described in Ref. [25]. Table 1 shows the comparison between the optical bandgap energy estimated from both set of samples, the ones of this paper (thin ones) and the ones of Ref. [25] (thick ones). From these data, we can see how the bandgap energy E_g_ increases from 1.73 eV to 2.56 eV according to the rise in Al mole fraction from x = 0 to 0.56, as summarized in Table 1.

It has to be noted that the high values of E_g_ are related to the so-called Burstein-Moss effect, i.e., the band filling of the conduction band and the blue shift of the bandgap energy of the material due to an increase of the unintentional doping (residual carrier concentrations close to 10^21^ cm^−3^) caused by impurities like oxygen and defects like nitrogen vacancies.

**Figure 4:**
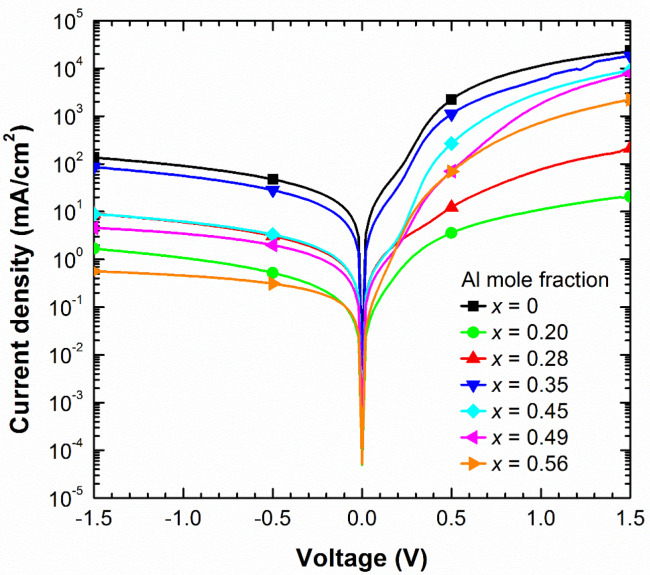
Current density vs voltage curves in the dark of the Al_x_In_1−x_N on Si (100) devices vs the Al mole fraction x.

Current density vs voltage (J-V) curves of the processed devices were measured in the dark to analyse the electrical behaviour of the diodes. Figure 4 illustrates the J-V curves of the fabricated n-p junctions with structures D1–D7 under dark conditions pointing to a rectifying behaviour. The reverse saturation current density (J_0_), series and shunt resistances (R_s_, R_sh_) and the diode ideality factor (η) were estimated fitting experimental data to the expression: J = J_0_ · [exp (V_d_/(η · V_T_)) − 1] + V_d_/R_sh_, where V_d_ is the diode voltage (V_d_ = V – J · R_s_), and V_T_ is the thermal voltage given by V_T_ = k_B_ · T/q (26 meV at room temperature).

From the results summed up in Table 2, we deduce that R_s_ increases from ~4.7 Ω·cm^2^ for InN- until ~60.3 Ω·cm^2^ for Al_x_In_1−x_N-based devices (x = 0.56), probably due to the high-top n-contact resistivity present at high Al mole fractions (see Figure 1c). The gradual increase of R_sh_ of more than two orders of magnitude (R_sh_ ~ 12–3100 kΩ·cm^2^) and the drop of the reverse saturation current density (J_0_ ~ 3.6−0.15 mA/cm^2^) with the Al mole fraction are assigned to the improved material quality of the Al_x_In_1−x_N layers compared to their InN counterparts, as previously demonstrated by our group [28]. At the same time, the ideality factor η drops from ~6.0 for devices with an Al mole fraction of 0.28 to ~3.0 for higher ones.

**Figure 5:**
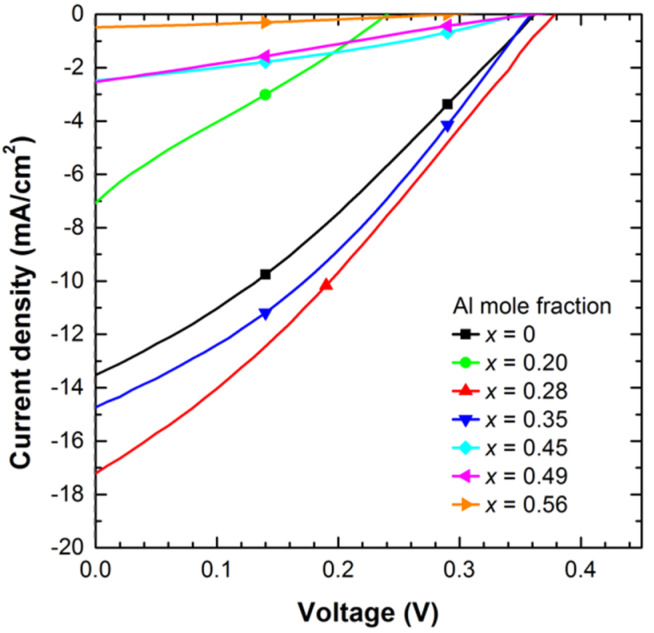
Current density vs voltage curves of the Al_x_In_1−x_N on Si (100) devices under AM1.5G illumination (1 sun) vs the Al mole fraction x.

Figure 5 presents the J-V measurements of all devices under AM1.5G illumination (1 sun) carried out to evaluate their photovoltaic performance in terms of open-circuit voltage (V_oc_), short-circuit current density (J_sc_), fill factor (FF), and power conversion efficiency (Eff.). J_sc_ was calculated taking into account the net area of the devices without the shadow of the top contact fingers of ~0.13 cm^2^.

The analysis of the photovoltaic parameters V_oc_, J_sc_, FF and efficiency extracted from the J-V curves of Figure 5 is summarized in Table 2 as a function of the Al mole fraction x. This analysis reveals that the open-circuit voltage, the short-circuit current density and the FF increase from 0.36 V to 0.39 V, from 13.4 mA/cm^2^ to 17.1 mA/cm^2^ and from 30.7% to 31.4% for InN and Al_0.28_In_0.72_N-based devices, respectively. This improvement leads to a maximum conversion efficiency of the Al_x_In_1−x_N on Si devices of 2.2% with 28% of Al content.

However, the high serial resistance obtained for devices growth under an Al power supply of 100 W (D2) leads to a low open-circuit voltage and short-circuit current values. This effect may be attributed to some alloy disorder on the nitride/Si interface that can be found at low Al power supplies and degrades the electrical behaviour of the junction [33].

When increasing the Al mole fraction above 28% the V_oc_, J_sc_, and efficiency drop down to 0.29 V, 0.5 mA/cm^2^, and 0.05% for the Al_0.56_In_044_N-based device, respectively. This trend is contrary as expected from theory when assuming the positive influence of increasing the Al mole fraction of the alloy, hence the bandgap energy, on the open-circuit voltage and the short-circuit current of the device, as previously reported [27]. So, the drop of the photovoltaic performance of the junction for Al contents above ~30% is attributed to the deterioration of the electrical properties of the Al_x_In_1−x_N layer (resistivity increases and carrier concentration and mobility decrease with the Al content) that increases the serial resistance of the junction and the top contact resistance of the device.

**Figure 6:**
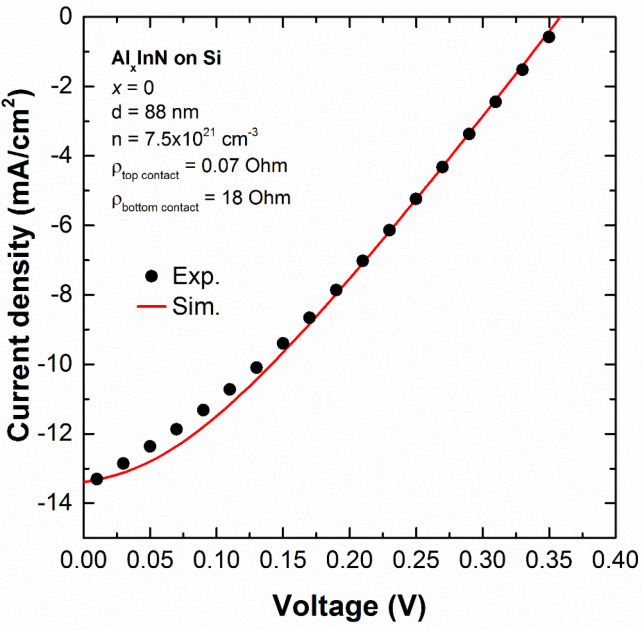
Comparison of the experimental J-V curve of the InN on Si device with the simulated one with PC1D program taking into account the simulation parameters from the inset.

To explore the theoretical limits of performance of these n-p junctions without the above-mentioned deterioration, we have performed simulations of the J-V curves under AM1.5G illumination (1 sun) of our devices with the PC1D program [34] following the procedure described in the paper [27]. First of all, we have simulated the InN-based device to have a starting point for the next simulations of the devices with increasingly Al mole fraction. As plotted in Figure 6, we have fitted the simulated J-V curve of the InN-based device to the experimental one, taking into account the thickness of the InN layer obtained by X-ray reflection measurements [28] and the n-type carrier concentration of the InN layer of Figure 2.

Afterwards, to study the evolution of the photovoltaic performance of the devices through their J-V curves with the Al mole fraction we have kept the thickness, doping and top and bottom contact resistances fixed to the values used for the simulation of the InN (see legend of Figure 6), while we have changed the following simulation parameters with the Al mole fraction: bandgap energy, electron affinity, refractive index, dielectric constant, intrinsic concentration, and Nc/Nv ratio. We note that the PC1D program offers the possibility to take into account the experimental absorption of the layers, so in our case, we have used the transmittance data measured in Al_x_In_1−x_N on sapphire samples grown at 300 °C with thicknesses above 300 nm and with Al mole fraction similar to those of this study [25]. From these simulations and under these particular conditions of nitride layer thickness and n-doping concentration, we can conclude that theoretically the photovoltaic performance of the Al_x_In_1−x_N on Si (100) devices would improve with the Al content as theoretically expected (higher V_oc_, J_sc_ and conversion efficiency), achieving a maximum of V_oc_ = 0.58 V, J_sc_ = 23.9 mA/cm^2^ and efficiency of 4.2% for n-Al_x_In_1−x_N on p-Si devices with 50% Al.

**Figure 7:**
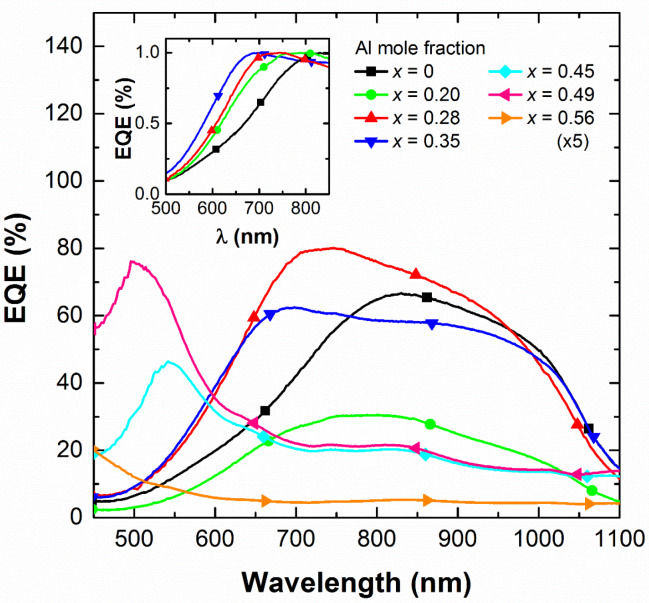
Influence of the Al mole fraction x on the EQE of the Al_x_In_1−x_N on Si (100) devices as a function of the wavelength. Inset: detail of the normalized EQE of devices with x ≤ 0.35.

To investigate the influence of the impinging wavelength on the photocurrent of the devices, responsivity measurements were carried out in the 375–1100 nm wavelength range. The external quantum efficiency (EQE) stands for the fraction of collected electron-hole pairs per incident photon and was estimated from these measurements as EQE = (J_op_/P_op_)·(hc/qλ), where J_op_ is the photocurrent density, P_op_ the optical power density impinging the device, q the electron charge, h is Planck’s constant, c is the speed of light and λ the wavelength of the incident light. The spectral evolution of the EQE curves of the devices is displayed in Figure 7. EQE values for all Al_x_In_1−x_N on Si devices at 860 nm are 66.7% (x = 0), are 29.9% (x = 0.20), 73.8% (x = 0.28), 58.3% (x = 0.28), 20.1% (x = 0.45), 21.4% (x = 0.49), and 1.1% (x = 0.56). The best device based on an Al_0.28_In_0.72_N on Si structure presents a maximum EQE of 80% at 750 nm.

For Al mole fractions below 0.35, the device EQE presents a broad spectral response covering the full solar spectrum from the visible to the near-infrared range with a peak in the visible above the Al_x_In_1−x_N bandgap energy followed by a photoresponse in the near-infrared with a cut-off ~1100 nm related to the Si band edge. Moreover, the band edge at short wavelengths follows the Al_x_In_1−x_N absorption shift with the Al mole fraction as plotted the normalized EQE in the inset of Figure 7.

However, for Al mole fractions above 0.35, the EQE drops accordingly with J-V results of Figure 7 without observing any contribution coming from the Si layer. This lessening of the photovoltaic performance of the devices is attributed to the increase of the Al_x_In_1−x_N resistivity of three orders of magnitude that lead to deterioration of the top contact performance. These results are very promising for Al_x_In_1−x_N on Si structures working as solar cells. Nevertheless, the degradation of the photovoltaic performance for devices with Al contents above 35% need to be overcome. For this aim, new strategies should be incorporated, such as doping the Al_x_In_1−x_N layer with Si and/or introducing a thin highly n-doped InN layer on top of the junction to improve the contact resistance. 

## 4. Conclusions

Photovoltaic devices based on n-Al_x_In_1−x_N on p-Si (100) hetero-junction solar cells with Al content ranging from 0% to 56% were fabricated by RF sputtering. The variation on the Al content leads to an evolution of the optical bandgap edge from 1.73 to 2.56 eV. Increasing the Al leads also to a 7-fold increase of the top n-contact resistance due to the higher Al_x_In_1-x_N resistivity and the drop of the n-type carrier concentration. Best results achieved for solar cells with x = 0.28 are V_oc_ = 0.39 V, J_sc_ = 17.1 mA/cm^2^, and FF = 31.4% under AM1.5G equivalent illumination (1 sun), pointing to a conversion efficiency of 2.12% without any top/back coating or surface treatment. For Al contents above 28 %, the photovoltaic performance is gradually reduced due to the strong top contact resistance, agreeing with the EQE of the devices, which drastically drops. Theoretical simulations of these structures point to a maximum of V_oc_ = 0.58 V, J_sc_ = 23.9 mA/cm^2^, and efficiency of 4.2% for n-Al_x_In_1−x_N on p-Si devices with 50% Al and a III-nitride resistivity in the ≈10^−4^ Ω·cm range.

## Figures and Tables

**Table 1:** Summary of the optical bandgap energy (E_g_) of the Al_x_In_1−x_N on Si devices.

Sample	P_Al_ (W)	*x*	E_g_ of Thin Samples (eV)	E_g_ of Thick Samples (eV)
D1	0	0	1.74	1.73
D2	100	0.20	1.87	-
D3	125	0.28	2.03	1.79
D4	150	0.35	2.08	2.13
D5	175	0.45	2.24	-
D6	200	0.49	2.38	2.27
D7	225	0.56	2.57	2.56

**Table 2:** Summary of the electrical performance of the Al_x_In_1−x_N on Si (100) devices. The area of the devices was estimated taking into account the area of the top n-contact of ~0.13 cm^2^.

Sample	x	Area (cm^2^)	R_s_ (Ω·cm^2^)	R_sh_ (kΩ·cm^2^)	J_0_ @-1V (µA/cm^2^)	η	V_OC_ (V)	J_SC_ (mA/ cm^2^)	FF (%)	Eff. (%)	EQE at 860 nm (%)
D1	0	0.67	4.7	12	3.60	2.8	0.36	13.4	30.7	1.49	66.7
D2	0.20	0.60	58.9	980	0.04	3.8	0.25	7.3	21.6	0.40	29.9
D3	0.28	0.62	2.1	180	0.41	6.0	0.39	17.1	31.4	2.12	73.8
D4	0.35	0.70	12.3	21	4.40	4.7	0.36	14.6	33.8	1.77	58.3
D5	0.45	0.60	14.1	180	0.31	4.2	0.35	2.5	32.8	0.28	20.1
D6	0.49	0.48	18.0	320	0.23	3.4	0.36	2.5	25.2	0.23	21.4
D7	0.56	0.65	60.3	3100	0.15	3.0	0.29	0.47	31.0	0.05	1.1
